# Giant Abdominal Pseudoaneurysm Secondary to Recurrent Pancreatitis: Imaging and Endovascular Intervention

**DOI:** 10.7759/cureus.32872

**Published:** 2022-12-23

**Authors:** Sheetal S Shelar, Rajasbala Dhande, Vadlamudi Nagendra, Manasa Suryadevara, Neha Shetty

**Affiliations:** 1 Radiodiagnosis, Jawaharlal Nehru Medical College, Datta Meghe Institute of Medical Sciences, Wardha, IND; 2 Radiology, Jawaharlal Nehru Medical College, Datta Meghe Institute of Medical Sciences, Wardha, IND

**Keywords:** coil embolization, angiography, doppler, pseudocyst, ct, ultrasonography, gastroduodenal artery, pancreatitis, pseudoaneurysm

## Abstract

Chronic or necrotizing pancreatitis is characterized by repeated inflammation of the pancreas, leading to multiple complications, a few of which are vascular, such as splanchnic venous thrombosis and arterial pseudoaneurysms. Even though the frequency of pseudoaneurysm formation in patients with pancreatitis is as high as 10%, there is not much importance given to its management in the radiologic literature. The splenic artery is the most common visceral artery affected by pseudoaneurysms, followed by the gastroduodenal and pancreaticoduodenal arteries. Usually, pseudoaneurysms occur due to the erosion of a peripancreatic or pancreatic artery into a pseudocyst, but this can also occur without the development of a pseudocyst. Pseudoaneurysms may be asymptomatic (usually the ones less than 5 cm), but some of them may pose a threat due to spontaneous rupture and subsequent fistulization into other organs. Therefore, early diagnosis and management are of prime importance. Here, in this article, we present a case of pseudoaneurysm of the gastroduodenal artery with characteristic imaging features and preferred, recent techniques of management.

## Introduction

Pseudoaneurysms of the peripancreatic and pancreatic arteries typically develop as a complication following chronic or necrotizing pancreatitis as a result of the arterial wall being broken down by proteolytic enzymes, trypsin being the most important of these [1]. The splenic artery (50%) is the most commonly involved artery, followed by the gastroduodenal (10%) and pancreaticoduodenal (10%) arteries. Spontaneous intraperitoneal rupture, followed by fistulization into the pancreatic duct and adjacent hollow organs, can be a fatal condition for the patient [2].

About 7.5% of pseudoaneurysms are asymptomatic and incidentally diagnosed. Giant pseudoaneurysms (more than 5 cm in diameter) may be symptomatic and be detected as a mass in the epigastrium that is pulsatile with referred pain in the left upper quadrant of the abdomen, accompanied by nausea and vomiting [3]. Multiple imaging modalities like ultrasound, doppler sonography, and computed tomography (CT) are used in the diagnosis, but angiography is the gold standard. It is also useful in the treatment, especially while considering endovascular embolization. Conventional management is via laparoscopic surgery, but over the years, alternatively, endovascular techniques have been employed and are slowly becoming the method of choice [4].

## Case presentation

A 26-year-old man presented with abdominal distention on and off in the left upper quadrant of the abdomen for three months. He had a previous history of pancreatitis and liver cirrhosis and was admitted for the same six months ago. His past history consisted of alcoholism for about 10 years with no other history of trauma, hypertension, or infective episode. His physical examination was unremarkable and his blood picture revealed RBC count values as 1.93 million/mm3 and hemoglobin as 2.8 G/dL. Serum lipase was slightly raised (237 U/L). The rest of the parameters were within normal limits.

**Figure 1:**
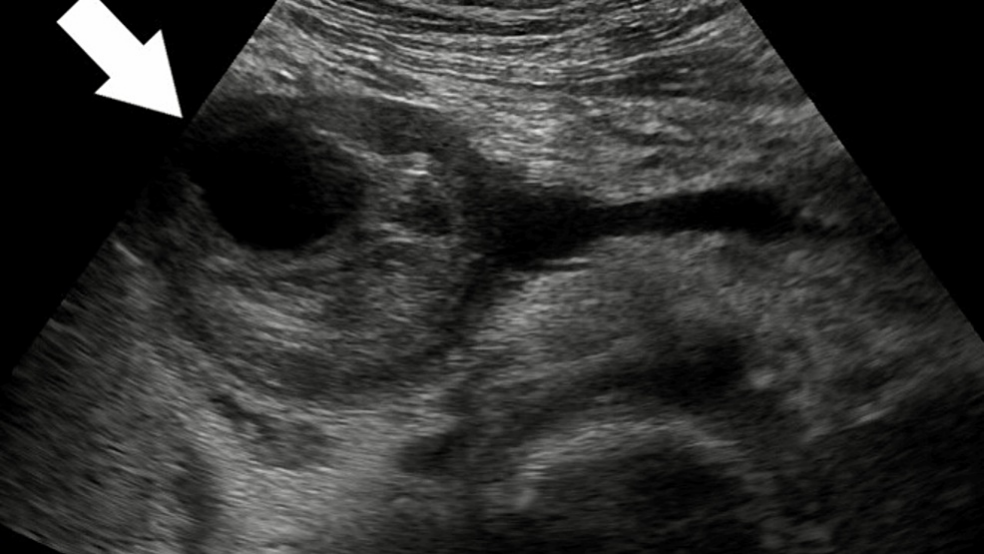
Greyscale US image showing a complex lesion with an internal lumen and thrombus surrounding it.

**Figure 2:**
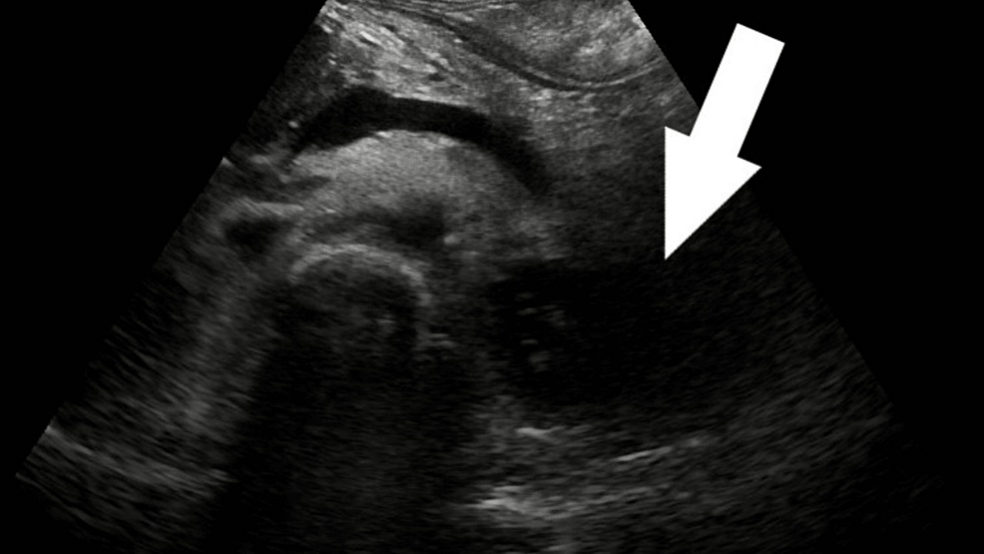
Greyscale US image showing fluid collection near the tail of the pancreas.

**Figure 3:**
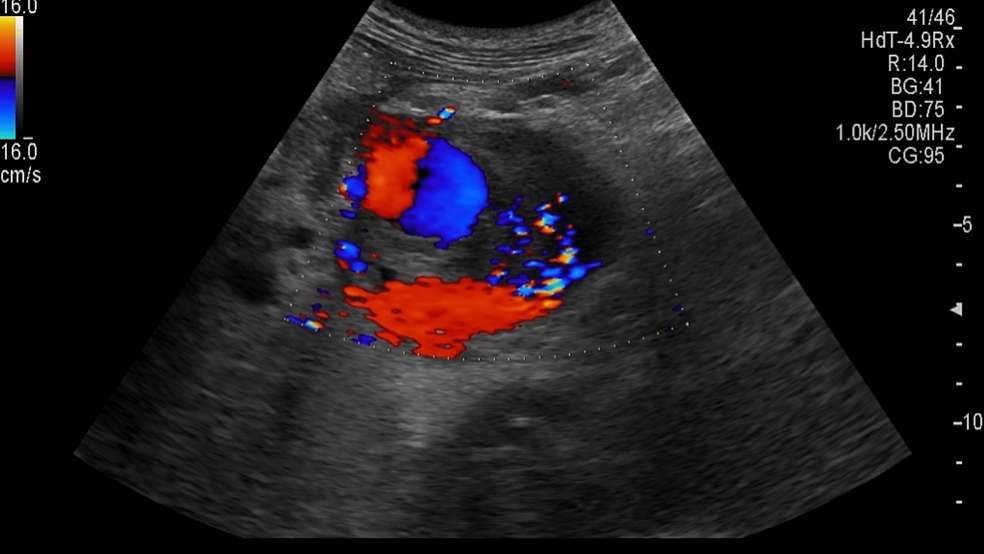
Colour Doppler of the lesion depicting the yin-yang sign of pseudo-aneurysm.

**Figure 4:**
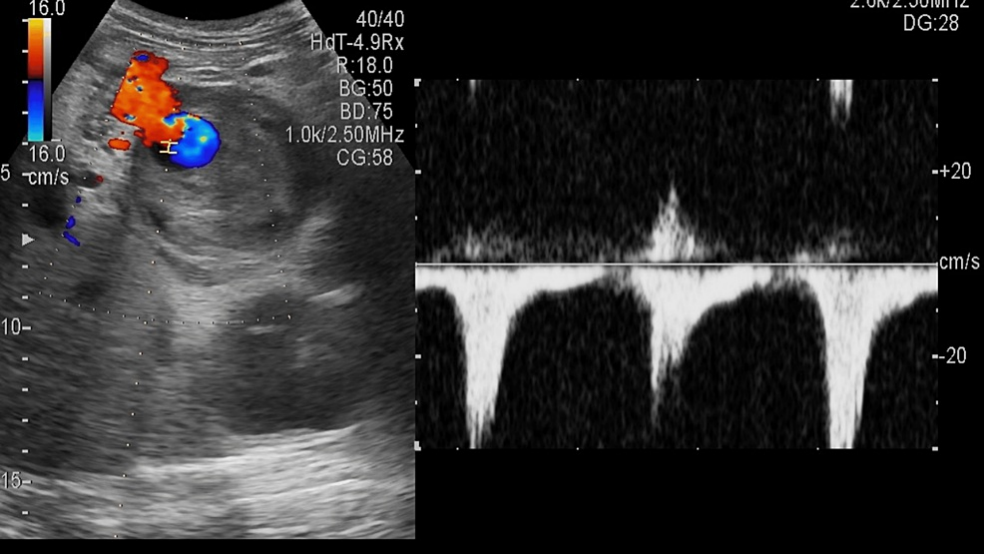
Pulsed Doppler of the affected artery showing to and fro waveform characteristic of pseudoaneurysm.

Ultrasound revealed a complex lesion of approximately 6.4 cm x 5.6 cm with a lumen and thrombus surrounding it (Figure 1). Some collection was seen around the tail of the pancreas/near the hilum of the spleen. The superior mesenteric artery and coeliac trunk appeared to be normal (Figure 2). Color Doppler showed a characteristic yin-yang sign suggestive of pseudo-aneurysm (Figure 3). Pulsed Doppler of the vessel showed a to and fro pattern of flow where 'to' reflected arterial blood entering the pseudo aneurysmal sac in the systolic cycle and 'fro' showed waveform blood moving out of the sac in the diastolic cycle (Figure 4).

**Figure 5:**
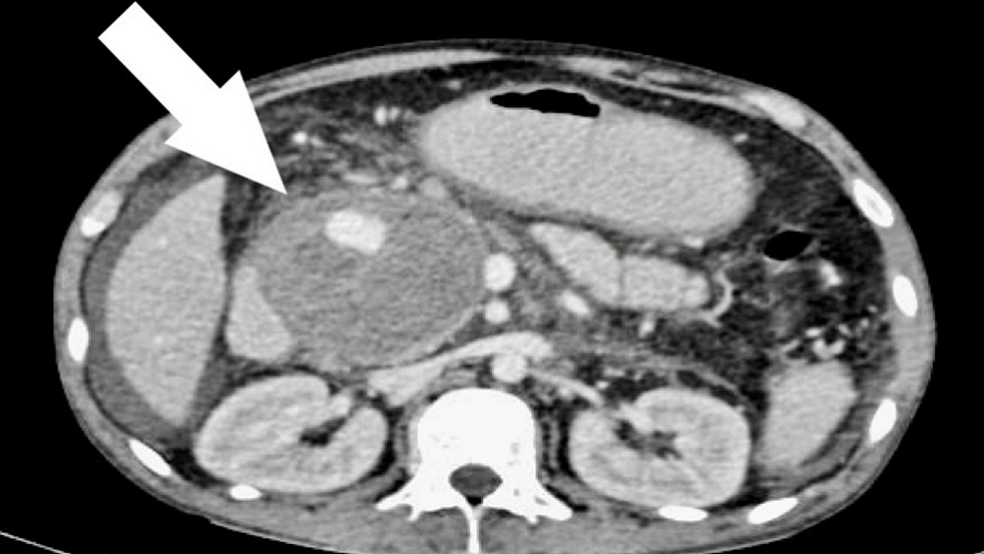
Contrast-enhanced CT axial section showing a well-defined cystic lesion measuring approximately 6.5 cm x 6 cm with an internal enhancing nidus close to the region of the gastroduodenal artery.

**Figure 6:**
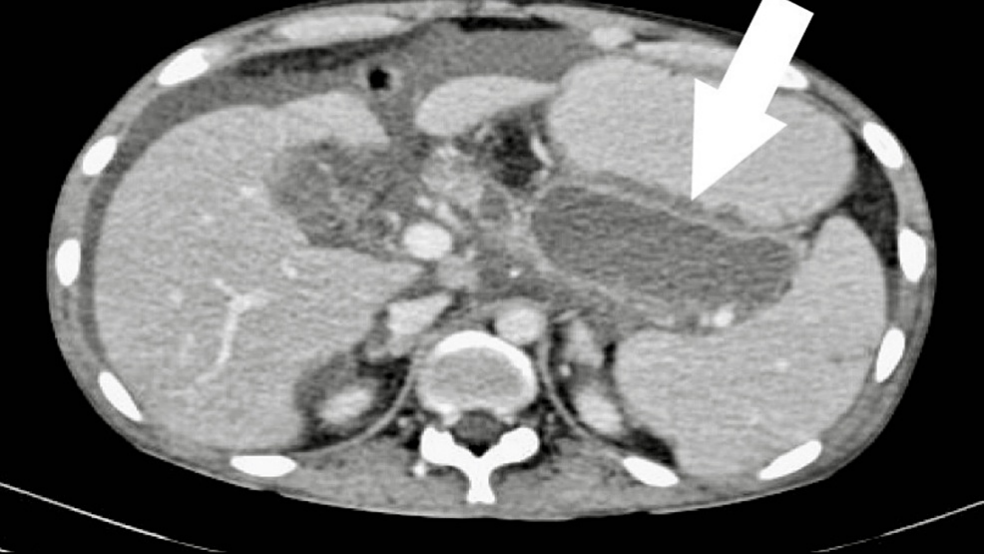
Contrast-enhanced CT axial section showing a well-defined cystic lesion around the tail of the pancreas with a thin enhancing wall.

Contrast-enhanced CT scan revealed a 6.5 cm x 6 cm well-defined cystic lesion with a 3 cm x 2.5 cm nidus that was enhanced similarly to the blood pool phase. This is in close relation to a small artery, possibly a branch of the gastroduodenal artery suggestive of gastroduodenal pseudoaneurysm. There was also evidence of mass effect in the form of compression of the duodenum (Figure 5). There was also evidence of an 11 cm x 4 cm cyst with enhancing wall in the region of the tail of the pancreas suggestive of a pancreatic pseudocyst (Figure 6). Peri-pancreatic lymph nodes, the largest being 17 mm, were also noted.

**Figure 7:**
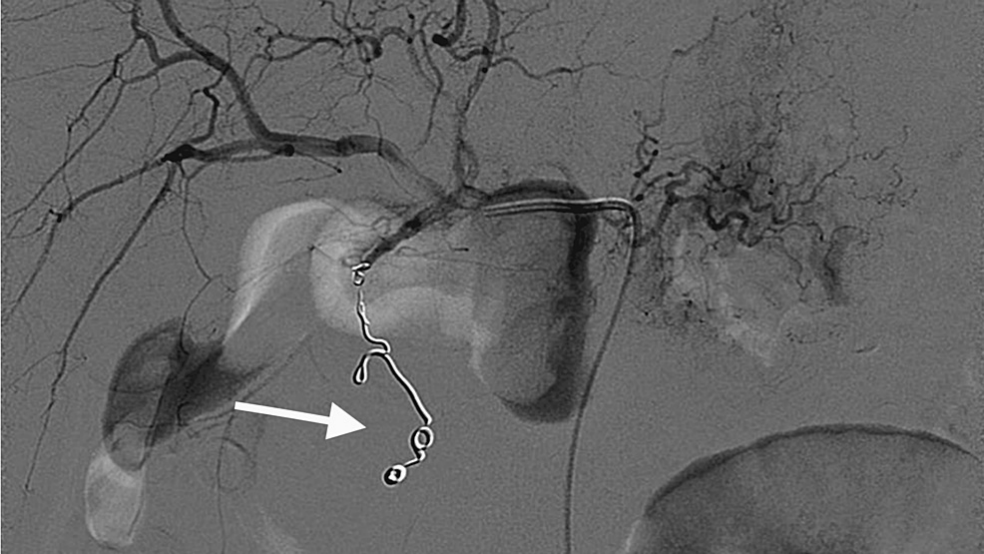
Digital subtraction angiography showing successful coil embolization of the gastroduodenal artery aneurysm (arrow) using a micro-catheter.

On CT angiography, successful coil embolization of the gastroduodenal artery and feeders to the aneurysm was done with an endovascular technique (Figure 7). Gastrointestinal visceral arterial embolization with a micro-catheter was performed.

Procedure

The surgery was carried out under all aseptic precautions. The right femoral artery access was obtained and a 5f sheath was inserted. The celiac trunk and gastroduodenal artery were cannulated using a 5f cobra catheter and selective cannulation with a microcatheter. An angiogram revealed the pseudoaneurysm and feeders from the branch of the gastroduodenal artery. Coil embolization of the gastroduodenal artery and feeders to the aneurysm was done using coils nester 35-14-6, nester 18-7-5, and hilal 18s-3.0-3. A repeat angiogram revealed occlusion of pseudoaneurysm and its feeders from the gastroduodenal artery (Figure 7). The 5f sheath was removed and hemostasis was achieved. The procedure was uneventful. 

## Discussion

Chronic alcohol consumption is the second most common cause of acute and chronic pancreatitis, respectively. Chronic pancreatitis develops from repeated attacks of acute pancreatitis. Complications such as pseudocysts, pancreatic duct strictures, and splenic vein thrombosis are more likely to occur. Pseudoaneurysm is not a well-known sequela but can sometimes occur within a pseudocyst. The splenic artery is the most commonly involved. Gastroduodenal artery pseudoaneurysm is the second most commonly encountered, even though its involvement is very rare overall [5].

Gastroduodenal artery aneurysms constitute approximately 1.5% of all visceral artery aneurysms [6] and are divided into true aneurysms and pseudoaneurysms based on the layers of the arterial wall involved. True aneurysms involve the destruction of all the arterial wall layers, whereas pseudoaneurysms are bound by only the tunica adventitia. Chronic or recurrent pancreatitis leads to thinning of the arterial wall due to the release of digestive enzymes like trypsin, leading to pseudoaneurysm formation. Pseudoaneurysms are therefore also susceptible to spontaneous rupture and fistulization into organs in the vicinity, which can be catastrophic. The occurrence of abdominal trauma is another important risk factor leading to the formation of a pseudoaneurysm. They rarely exceed 3 cm in diameter, and those greater than 5 cm are known as giant pseudoaneurysms. In the presented case, the pseudoaneurysm occurred as an independent sequela of recurrent pancreatitis, even though the pseudocyst was also a coincidental finding.

Ultrasonography followed by CECT or CT angiography is the usual algorithm, after which the decision of either endovascular embolization or laparoscopic surgery is taken. Surgery is usually the gold standard, with suture ligation of the vessel proximal and distal to the pseudoaneurysm as the preferred mode of management [4, 7]. In recent years, transcatheter endovascular embolization has taken over because of its advantages, namely low mortality and higher rates of successful embolization [8].

## Conclusions

Pseudoaneurysm formation is a potential complication in cases of pancreatitis and should be given importance because of the high rate of mortality when left untreated. Most pseudoaneurysms are asymptomatic and can symptomatically present with rupture and internal bleeding. Therefore, early diagnosis through imaging i.e., ultrasonography followed by CT, and subsequent appropriate management through surgical or endovascular techniques, should be considered.
